# Telomerase expression is sufficient for chromosomal integrity in cells lacking p53 dependent G_1 _checkpoint function

**DOI:** 10.1186/1477-3163-4-18

**Published:** 2005-10-06

**Authors:** Dennis A Simpson, Elizabeth Livanos, Timothy P Heffernan, William K Kaufmann

**Affiliations:** 1 Department of Pathology and Laboratory Medicine, Lineberger Comprehensive Cancer Center, and Center for Environmental Health and Susceptibility, University of North Carolina at Chapel Hill, CB 7295, Chapel Hill, NC 27599, USA

## Abstract

**Background:**

Secondary cultures of human fibroblasts display a finite lifespan ending at senescence. Loss of p53 function by mutation or viral oncogene expression bypasses senescence, allowing cell division to continue for an additional 10 – 20 doublings. During this time chromosomal aberrations seen in mitotic cells increase while DNA damage and decatenation checkpoint functions in G_2 _cells decrease.

**Methods:**

To explore this complex interplay between chromosomal instability and checkpoint dysfunction, human fibroblast lines were derived that expressed HPV16E6 oncoprotein or dominant-negative alleles of p53 (A143V and H179Q) with or without the catalytic subunit of telomerase.

**Results:**

Cells with normal p53 function displayed 86 – 93% G_1 _arrest after exposure to 1.5 Gy ionizing radiation (IR). Expression of HPV16E6 or p53-H179Q severely attenuated G_1 _checkpoint function (3 – 20% arrest) while p53-A143V expression induced intermediate attenuation (55 – 57% arrest) irrespective of telomerase expression. All cell lines, regardless of telomerase expression or p53 status, exhibited a normal DNA damage G_2 _checkpoint response following exposure to 1.5 Gy IR prior to the senescence checkpoint. As telomerase-negative cells bypassed senescence, the frequencies of chromosomal aberrations increased generally congruent with attenuation of G_2 _checkpoint function. Telomerase expression allowed cells with defective p53 function to grow >175 doublings without chromosomal aberrations or attenuation of G_2 _checkpoint function.

**Conclusion:**

Thus, chromosomal instability in cells with defective p53 function appears to depend upon telomere erosion not loss of the DNA damage induced G_1 _checkpoint.

## Background

Normal diploid fibroblasts proliferate in secondary cultures for a finite number of population doublings until a growth arrest known as replicative senescence, or M1, is reached [1]. This limitation in lifespan is believed to be due to the continuous shortening of the telomeres with each cell division [2]. Recent evidence has suggested that an alteration in the structure of one or more telomeres may, in fact, be what triggers cells to enter replicative senescence, a permanent p53-dependent G_1 _arrest [3,4]. Regardless of the exact trigger of senescence, inactivation of p53 allows cells to bypass senescence and continue to divide until a second growth restriction termed crisis, or M2, is reached [5]. Cells in crisis contain numerous structural and numerical chromosomal abnormalities which may be due to cycles of chromosome fusion (dicentric chromosomes) and subsequent resolution of the fusion (chromosome break) during mitosis [2]. A previous study has demonstrated that during the phase of extended proliferation after bypass of M1, telomeres in p53-defective, telomerase-negative cells can erode to the point where little or no telomeric repeat DNA can be detected [6]. Chromosomes without telomeres appear to be substrates for DNA repair pathways resulting in telomere associations and formation of dicentric and ring chromosomes. The resolution of these unstable structures is believed to result in the other structural and numerical abnormalities in chromosomes observed in cells in crisis (i.e., breaks, exchanges, aneuploidy, polyploidy).

Prevention of telomere erosion by ectopic expression of the catalytic subunit of human telomerase (hTERT) has been shown to prevent crisis in cells expressing SV40 large T antigen or HPV16E6 oncoprotein [7-10]. Normal diploid human fibroblasts expressing hTERT have been reported to maintain a normal karyotype and preserve cell cycle checkpoint function for at least 200 population doublings [11,12], although others have suggested that otherwise normal telomerase-expressing human fibroblasts do display alterations in expression of tumor suppressor genes, growth characteristics, and transient genetic instability [13-16] These studies have failed to directly address the question as to whether cells can maintain a stable genome in the absence of a functional DNA damage induced G_1 _checkpoint. Here we report that in the absence of telomere erosion cells defective for p53 signaling can maintain stable genomes for >175 population doublings. This study found that normal diploid cells expressing hTERT maintain a normal karyotype for at least 100 PD's but eventually did become numerically abnormal. We also report that two independent p53-defective lines which emerged from crisis by reactivation of telomerase displayed remarkably stable karyotypes.

## Materials and methods

### Plasmids and viruses

**Figure 8:**
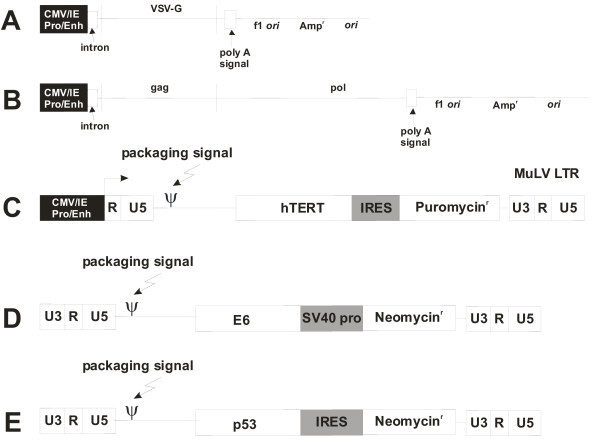
Expression constructs used in this study. CMV/ie Pro/Enh, Cytomegalovirus immediate early promoter/enhancer sequence; VSV-G, vesicular stomatitis virus glycoprotein G; SV40 Pro, simian virus 40 promoter/origin sequence; IRES, internal ribosome entry sequence; MuLV LTR, murine leukemia virus long terminal repeat; hTERT, human telomerase catalytic subunit. A) Plasmid pCI VSV-G expressing VSV-G used to pseudotype replication-defective retrovirus particles. B) Helper plasmid pCI GPZ for packaging replication-defective retrovirus particles. C) pDSWK-8 plasmid vector used to package telomerase cDNA. D) pLXSN-E6 plasmid vector for packaging HPV16 E6. E) pLXIN+p53-A143V and pLXIN+p53-H179Q plasmid vectors for packaging dominant-negative p53 alleles.

All cloning steps were carried out according to standard methods [17]. Plasmids were maintained in the DH5α strain of *E. coli*. Replication-defective retroviruses used in this study and helper plasmids for packaging are shown in Figure 8. The hTERT retroviral expression vector, pDSWK-8, was created by cloning the hTERT cDNA from pBABE/Hyg-hTERT (Dr. Robert A. Weinberg, Whitehead Institute for Biomedical Research) into the EcoRI and HpaI sites of the pHIT-2 retroviral backbone (Dr. John Olsen, University of North Carolina). cDNA's encoding an alanine to valine substitution at amino acid 143 or a histidine to glutamine substitution at amino acid 179 in p53 (p53-A143V and p53-H179Q respectively) were provided by Drs. David Wynford-Thomas (University of Aberdeen) and Dr. Howard Liber (Massachusetts General Hospital), respectively. Retroviral expression vectors containing these dominant-negative forms of p53 were constructed by cloning the cDNA into the EcoRI site of the pLXIN (Clonetech) retroviral expression vector. The pLXSN-E6 retroviral expression vector containing the HPV16 E6 oncoprotein DNA was a gift from Dr. Denise Galloway (Fred Hutchinson Cancer Center). Vesicular stomatitis virus glycoprotein G-pseudotyped, replication-defective retroviruses were produced as previously described following transient transfection of viral vector and helper plasmids into HEK 293T cells [18-20]. Transfections of plasmids for virus production were done using Superfect™ or Polyfect™ (Qiagen) according to the manufacturer's protocol.

### Cell Culture

A normal human fibroblast strain designated NHF1 was derived from neonatal foreskin as previously described [21]. All cell culture, including retroviral production, was performed in a humidified, water-jacketed incubator at 37°C with a 5 % CO_2 _atmosphere. NHF1 cells and all cell lines derived from the parental NHF1 secondary culture were maintained in MEM (Gibco Invitrogen Corp.) supplemented with 10 % defined fetal bovine serum (Hyclone), 2 mM L-glutamine (Gibco Invitrogen Corp.), and 100 μM non-essential amino acids (Gibco Invitrogen Corp.). HEK 293T cells were maintained in DMEM-H (Gibco Invitrogen Corp.) supplemented with 10 % defined fetal bovine serum (Hyclone), 2 mM L-glutamine (Gibco Invitrogen Corp.), 100 μM non-essential amino acids (Gibco Invitrogen Corp.), and 20 mM HEPES pH 7.3 (Sigma Chemical Co.). Transductions were carried out according to standard methods as described previously [22]. Cells at passages 5 or 6 were simultaneously transduced with both the hTERT-expressing virus and one of the viruses disrupting p53 function and/or empty vectors to derive the cell lines listed in Table 1. At the time of transduction, the NHF1 cells were estimated to have undergone 15 – 20 population doublings *in vitro*. Transductants were selected by 2 weeks growth in media containing 300 ng/ml puromycin (Sigma Chemical Co.) plus 200 μg/ml of active G418 (Gibco Invitrogen Corp.) and, following this initial selection, lines were maintained without antibiotics. Cells were seeded each passage at a density of 5300 – 5500 cells per cm^2^. The population doubling level (PDL) of the culture was defined as the sum of the population doublings (PD) of each passage. The PD of each passage was determined using the following equation:

**Table 1:** Status of p53 in Cell Lines

Cell Line	hTERT	p53 Protein
F1-hTERT+LXIN	+	WT^1^
F1-hTERT+p53-A143V	+	WT/DN^2^
F1-hTERT+p53-H179Q	+	WT/DN
F1-hTERT+E6	+	-
F1-HIT+LXIN	-	WT
F1-HIT+p53-A143V	-	WT/DN
F1-HIT+p53-H179Q	-	WT/DN
F1-HIT+E6	-	-

1. WT: Wild Type

2. DN: Dominant Negative



Cell lines were monitored for mycoplasma contamination using the Gen-Probe kit (Gen Probe Inc. San Diego CA) according the manufacturer's instructions. By this method the cell lines remained free of mycoplasma for the duration of the study.

### Cell Cycle Checkpoint Analysis

DNA damage checkpoint responses were assessed following exposure to 1.5 Gy of IR from a ^137^Cs source (GammaCell 40, MDS Nordion, Canada) at a dose-rate of 86 rads per minute. G_1 _checkpoint function was assessed by measuring 5-bromo-2'-deoxy-uridine (BrdU, Sigma Chemical Co.) incorporation from six to eight hours following exposure to 1.5 Gy as previously described [23-25]. Flow cytometric and microscopic determination of mitotic indices were shown to yield equivalent results [26,27]. DNA damage G_2 _checkpoint function was assessed by determining the mitotic index of cultures two hours following irradiation. Mitotic index was determined using flow cytometry to measure the number of cells expressing the phospho-histone H3 mitotic epitope or by directly counting Giemsa- or DAPI-stained mitotic figures as previously described [28-30] Spindle damage checkpoint function was assessed by seeding cells into medium containing 100 ng/ml colcemid (Sigma Chemical Co.) for 24 or 48 hours. The cells were labeled with BrdU during the last two hours of this incubation. Cells were then analyzed by flow cytometry as described above to determine the percentage of cells with >4n DNA content.

### Chromosomal Analyses

Metaphase spreads were prepared from the 8 cell lines listed in Table 1 at the earliest possible PDL following selection and then every 10 – 20 PD thereafter until telomerase-negative cells reached crisis. All metaphase preparations were done according to previously described methods [6]. Fifty metaphases from each cell line at each PDL were analyzed and scored for number of chromosomes, and the numbers and types of structural abnormalities. G-banding was done according to standard protocols [31] and representative karyotypes were assembled after analysis of 20 to 25 metaphases.

### Western Blot Analysis

Logarithmically growing cells were seeded at 5 × 10^5 ^per 100-mm dish and incubated for 48 hr. Cultures were irradiated as described above and incubated for 6 hr at 37°C. Cells were harvested by trypsinization, washed once in PBS, and resuspended in lysis buffer (100 mM sodium phosphate buffer, pH 7.2, 10 mM EDTA, 10 mM EGTA, 1.5 M NaCl, 10% NP40, supplemented with 10 mM 4-(2-aminoethyl) benzenesulfonyl fluoride (AEBSF, Sigma Chemical Co.), 10 mM β-glycerophosphate (Sigma Chemical Co.), 10 mM sodium orthovanadate (Sigma Chemical Co.), and 10 ug/ml of leupeptin (Sigma Chemical Co.) and aprotinin Sigma Chemical Co.). Protein concentrations were determined using the Bio-Rad D_*C *_Protein Assay (Bio-Rad Laboratories) according to the manufacturer's protocol. Samples containing 100 μg protein were mixed with an equal volume of 2 × Laemmli sample buffer (125 mM Tris-HCl, pH 6.8, 4% SDS, 20% glycerol) containing 5% β-mercaptoethanol (Sigma Chemical Co.), boiled, and separated by SDS-PAGE. Proteins were transferred to nitrocellulose and probed with antibody against p21^Waf1^(Neomarkers) and detected with goat anti-rabbit HRP using the ECL substrate (both Amersham Pharmacia Biotech).

## Results

### Validation of Cell Lines

**Figure 1:**
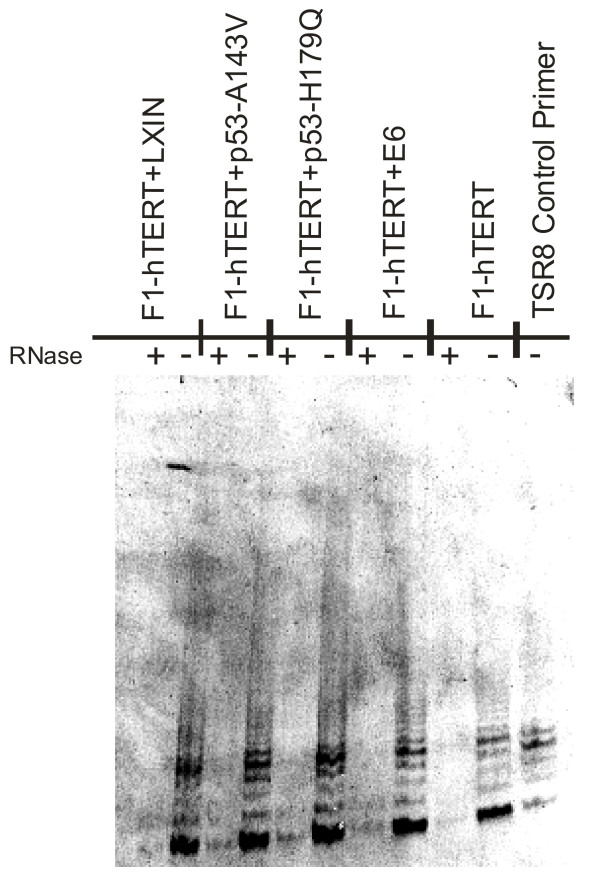
Assessment of telomerase activity in cell lines transduced with DSWK-8 by TRAP assay. Each of these cell lines exhibits an RNase-sensitive PCR product.

This study utilized eight isogenic cell lines differing only in expression of telomerase and p53 function as listed in Table 1. Following selection the cells transduced with DSWK-8 (F1-hTERT lines) were assayed for telomerase expression by TRAP assay [32-34]. As shown in Figure 1, all cell lines transduced with DSWK-8 were telomerase-positive. Cell lines transduced with the empty telomerase vector (HIT) were telomerase-negative (data not shown). Western immunoblot analysis confirmed there was significant overexpression of p53 in lines expressing the p53-V143A and p53-H179Q alleles, and no detectable p53 in lines expressing HPV16E6 (not shown).

**Figure 2:**
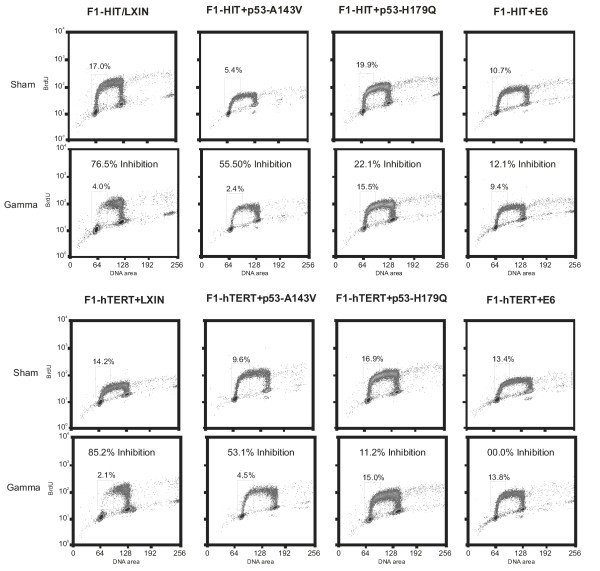
G_1 _checkpoint analysis of cell lines. Cells at population doubling level 25 – 30 were tested for G_1 _checkpoint function. Incorporation of BrdU was analyzed 6 – 8 hours after exposure to 1.5 Gy. The radiation-induced reduction in the percentage of cells in the first half of S-phase was determined as a quantitative measure of G_1 _checkpoint function.

The ability of the cells to delay entry into S-phase following exposure to 1.5 Gy of ionizing radiation (IR) was assessed as a quantitative index of p53-dependent G_1 _checkpoint function (Figure 2). The F1-HIT+LXIN and F1-hTERT+LXIN cell lines that have an intact p53 signaling pathway displayed an effective G_1 _checkpoint response to DNA damage. In this line the percent of cells in the first half of S phase 6 – 8 h after irradiation was reduced by >75% due to a G_1 _arrest. Cells transduced with HPV16E6 and p53-H179Q exhibited severely attenuated G_1 _checkpoint function. Less than 15% of HPV16E6-expressing cells were delayed in G_1 _while cells expressing p53-H179Q had <25% arrested in G_1 _post-irradiation. Cell lines expressing the p53-A143V dominant-negative form of p53 retained approximately half of the normal G_1 _checkpoint response with about 50% of irradiated cells delayed in G_1_. Expression of telomerase had no effect on the radiation-induced G_1 _arrest.

**Figure 3:**
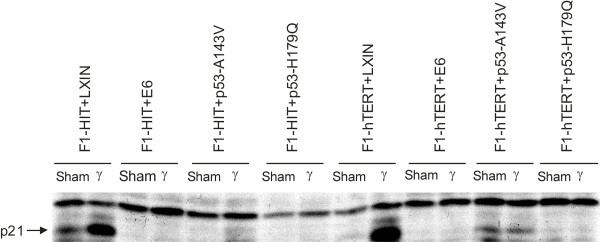
Assessment of p53-dependent induction of p21^Waf1 ^following IR. Western immuno blot to ascertain p21^Waf1 ^induction 6 hr following 1.5 Gy IR was done as described in Methods.

Immunoblot analysis of p21^Waf1 ^expression confirmed the biological analysis of the G1 checkpoint (Figure 3). Cell lines transduced with the empty LXIN vector expressed p21^Waf1 ^in sham-treated controls and expression was induced after treatment with IR. Lines expressing HPV16E6 and p53-H179Q, which displayed severe attenuation of G_1 _checkpoint function, did not express p21^Waf1 ^in sham-treated controls nor after irradiation. Lines expressing p53-A143V did not display full ablation of expression or induction of p21^Waf1 ^as evident by the low level of expression in sham-treated controls and some induction of protein after irradiation. As was the case for radiation-induced G_1 _arrest, expression of telomerase did not affect the expression or induction of p21^Waf1^. Expression of HPV16E6 and p53-H179Q ablated expression of p21^Waf1 ^and induced a severe attenuation of G_1 _checkpoint function, while expression of p53-A143V attenuated expression of p21^Waf1 ^while reducing G_1 _checkpoint function by about 50%. Thus the p53-A143V lines displayed only a partial loss of p53 function.

**Figure 4:**
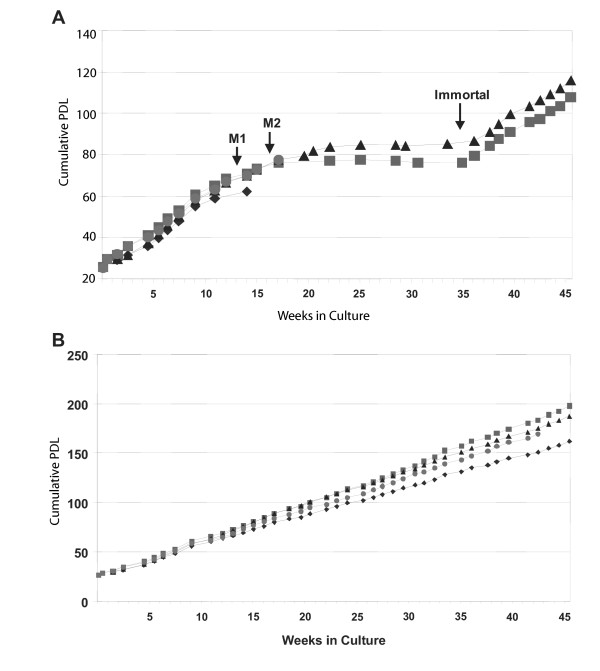
Growth curves of transduced fibroblasts. The x-axis is the number of weeks of continuous culture. The y-axis represents the number of population doublings the cultures had accumulated. A. (◆) F1-HIT+LXIN; (■) F1-HIT+p53-A143V; (▲) F1-HIT+p53-H179Q; F1-HIT+E6. The empty vector control (●) F1-HIT+LXIN cell line underwent senescence at PDL 61 while the cells with defective p53 function continued to divide for an additional 10 – 20 population doublings. At this point cell death equaled cell division, resulting in no net gain of cell number over time. During the approximate 16-week duration of this phase the E6-expressing cells died. B. Continuous growth of hTERT-expressing lines. (◆) F1-hTERT+LXIN; (■) F1-hTERT+p53-A143V; (●) F1-hTERT+p53-H179Q; F1-hTERT+E6.

In contrast to their differing responses in the DNA damage G_1 _checkpoint the non-telomerized F1-HIT+p53-A143V line behaved like the non-telomerized F1-HIT+HPV16E6 and F1-HIT+p53-H179Q lines and bypassed the replicative senescence checkpoint during *in vitro *aging. Cell population expansion was monitored continuously and all of the telomerase-negative lines initially displayed equivalent growth *in vitro *(Figure 4A). Cells expressing HPV16E6 or the dominant-negative alleles of p53 continued to grow for 15 – 20 population doublings beyond the 60 PDL at which F1-HIT+LXIN senesced and arrested growth (Figure 4A). After PDL 78 cell death exceeded cell birth in the telomerase-negative, E6-expressing culture, and the culture died by what is classically known as telomere crisis [35]. Although population doublings did not increase beyond PDL 80 – 85 in the p53-A143V and p53-H179Q lines for a period of about 18 weeks and the cells appeared to be in crisis, viable cells nevertheless remained on dishes. After 36 weeks in culture, population expansion resumed and two immortal lines were recovered. The behavior of the cell lines was similar to that detailed previously [25] with p53-effective, telomerase-negative fibroblasts undergoing replicative senescence after 60 population doublings, and the p53-defective, telomerase-negative lines bypassing senescence and then undergoing telomere crisis.

**Figure 5:**
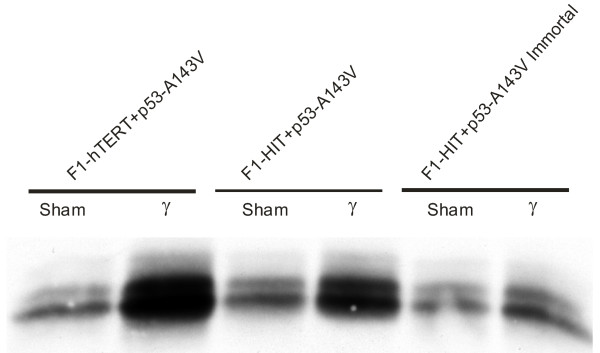
p53-A143V-expressing cells are able to partially induce p21^Waf1^. Western-immuno blot to demonstrating p21^Waf1 ^induction in p53-A143V-expressing cells. Log-phase cultures were irradiated with 1.5 Gy. Six hours post-irradiation, the cells were harvested and lysed in loading buffer.

Population expansion in the lines transduced directly with hTERT was continuous and equivalent to that seen in telomerase-negative lines at PDL 40 – 60 and in the spontaneously immortalized lines at PDL 90 – 110 (Figure 4B). The hTERT-expressing lines were carried to PDL >175 without reduction in growth rate. P53-dependent G_1 _checkpoint function was monitored during the various phases of cellular aging *in vitro *and found not to vary substantially (Table 2). Additionally the spontaneously immortalized cell line expressing the p53-A143V dominant negative p53 allele was still able to induce a small amount of p21^Waf1 ^following exposure to 1.5 Gy (Figure 5)

**Table 2:** G_1 _Checkpoint Function of Aging Cell Lines

	**% of Cells Exhibiting a G**_**1 **_**Delay**							
	
**PDL**^**1**^	**F1-hTERT+LXIN**	**F1-hTERT+p53-A143V**	**F1-hTERT+p53-H179Q**	**F1-hTERT+E6**	**F1-HIT+LXIN**	**F1-HIT+p53-A143V**	**F1-HIT+p53-H179Q**	**F1-HIT+E6**
30 – 35	100	51	11	0	77	56	22	12
40 – 50	94	44	20	6	95	55	29	0
60 – 65	92	65	5	7	85	54	10	5
75 – 85	80	57	17	1				
>100^2^	97	66	0	0		37^3^	0^3^	

Avg.^4^	93 ± 8	57 ± 9	11 ± 8	3 ± 3	86 ± 9	55 ± 1	20 ± 10	6 ± 6

1. Population Doubling Level

2. PDL of hTERT expressing lines 190 – 210

3. Spontaneous Immortalized Derivative

4. Average ± standard deviation. Average for HIT lines excludes spontaneous immortals.

**Figure 6:**
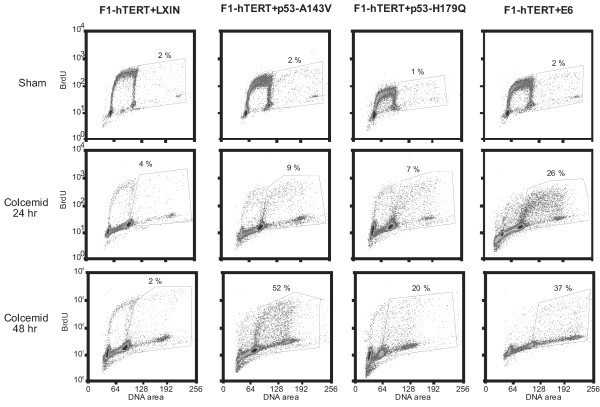
Assessment of spindle damage checkpoint function in p53-defective fibroblast lines. Endoreduplication was assessed by flow cytometric analysis of BrdU-labeled cells 24 and 48 h after addition of colcemid to culture medium.

A previous report indicated that some dominant-negative p53 alleles induced a gain of function [36]. This gain of function was identified using a "spindle damage" assay that measures the ability of cells to become polyploid when incubated with microtubule poisons such as colcemid. This phenomenon was examined in the four-telomerized cell lines derived for this study. Following 24 or 48 hours incubation in 100 ng/ml colcemid, cells were labeled for two hours with BrdU and then analyzed by flow cytometry to determine the rate of DNA synthesis in diploid and tetraploid nuclei. As shown in Figure 6, all cell lines with defective p53 signaling underwent endoreduplication and displayed increased frequencies of tetraploid S-phase cells when incubated in colcemid. The isogenic F1-hTERT+LXIN line with effective p53-dependent G_1 _checkpoint function did not display this endoreduplication when incubated with colcemid. Thus, inactivation of p53 expression with HPV16E6 oncoprotein induced the same susceptibility to endoreduplication during incubation in colcemid as was seen using dominant-negative mutant p53 alleles to disrupt p53 signaling.

### Chromosomal Instability

An assessment of chromosomal integrity was done on all eight cell lines within five population doublings of gene transduction to assess the background level of structural and numerical abnormalities in the population and then at PDL 60 (normal replicative senescence point), and PDL 75 – 85 (crisis). Table 3 demonstrates that there were no differences among the various cell lines at the first PDL examined. As was previously observed upon transduction of telomerase-negative fibroblasts with HPV16E6 [6], soon after inactivation of p53 with the dominant-negative alleles, chromosomal number and structure appeared normal. However, as the lines aged and approached the normal replicative senescence point of 60 population doublings, the number of metaphases exhibiting structural and numerical abnormalities increased dramatically in the telomerase-negative cell lines with defective p53 function. At PDL 76 – 77, the majority of metaphases derived from the telomerase-negative HPV16E6-, p53-A143V-, and p53-H179Q-expressing cells exhibited hypodiploidy and/or dicentric chromosomes. The p53-defective lines that were transduced with hTERT to express telomerase did not display these aging-related instabilities in chromosome numbers and structure.

**Table 3:** Quantification of Structural and Numerical Chromosomal Abnormalities in Cell Lines

		**% of Metaphases Containing:**									
		
**Cell Line**	**PDL**^**1**^	**# Chromosomes**									
							
		**≤ 44**	**45 – 47**	**48 – 85**	**86 – 99**	**≥ 100**	**Dicentrics + Rings**	**TA**^**2**^	**Breaks**	**Fragments**	**Other**^**3**^
F1-hTERT+LXIN	32	9	85	0	7	0	0	0	2	0	0
	60	2	93	2	3	0	0	2	2	0	0
	76	7	93	0	0	0	0	0	0	0	0

F1-hTERT+p53-A143V	31	5	86	3	5	2	0	3	0	0	0
	66	0	90	4	4	2	2	0	2	0	0
	85	0	96	2	2	0	0	5	4	0	0

F1-hTERT+p53-H179Q	33	10	88	0	2	0	0	0	0	0	0
	63	2	87	2	9	0	0	0	2	2	0
	84	2	98	0	0	0	0	0	0	0	0

F1-hTERT+E6	30	0	86	4	11	0	0	0	0	2	0
	63	2	93	4	2	0	4	0	0	0	0
	84	2	94	2	0	0	2	0	2	0	0

F1-HIT+LXIN^4^	31	10	85	2	2	2	0	0	0	0	0
	59	2	97	0	2	0	5	0	0	0	0

F1-HIT+p53-A143V	32	8	89	0	4	0	0	0	4	0	0
	65	27	48	10	10	5	53	2	3	5	7
	76	44	32	7	11	4	22	2	2	20	0

F1-HIT+p53-H179Q	33	5	91	2	0	0	0	0	0	2	2
	63	7	76	2	15	0	37	4	2	7	2
	77	23	62	4	9	0	38	2	2	8	2

F1-HIT+E6	32	7	79	9	4	2	5	2	2	2	2
	63	33	60	2	5	0	43	0	3	7	0
	77	66	24	0	5	0	39	10	0	24	0

1. Population Doubling Level

2. Telomere Associations

3. Exchange Aberrations + Deletions

4. These cells senesced at 60 population doublings

### Attenuation of DNA damage G_2 _checkpoint function

**Figure 7:**
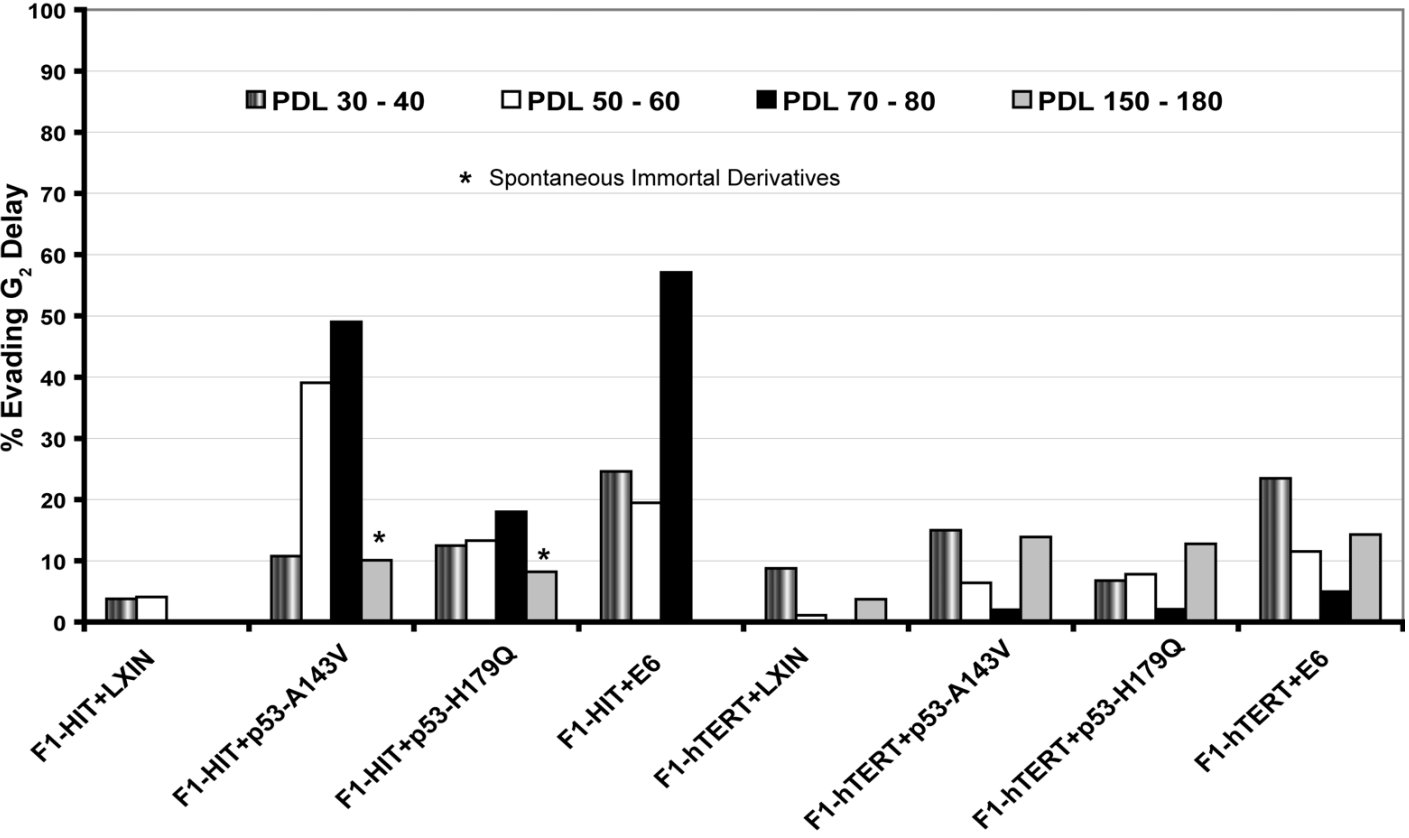
DNA damage G_2 _checkpoint function in aging fibroblasts. Log-phase cultures at low and high PDL were treated with 1.5 Gy of IR then incubated for 2 h before determination of mitotic index. The percentage of mitotic cells in irradiated cultures was divided by the percentage of mitotic cells in sham-treated controls to determine the percent of cells evading G_2 _delay [25].

Previous studies from this laboratory have demonstrated that DNA damage G_2 _checkpoint function becomes attenuated in congruence with chromosomal instability [6,25,29]. Figure 7 depicts DNA damage G_2 _checkpoint function in the cell lines at various *in vitro *PDLs. The F1-HIT+LXIN and F1-hTERT+LXIN lines displayed a typically effective G_2 _checkpoint response with on average >95% of G_2 _cells being delayed in their entry to mitosis after treatment with 1.5 Gy. Expression of the dominant-negative p53 alleles and HPV16E6 induced a modest attenuation of G_2 _checkpoint function measured at PDL 30 – 40 with 7 – 24% of p53-defective cells evading radiation-induced G_2 _delay. The telomerase-negative lines expressing p53-A143V and HPV16E6 displayed further severe attenuation of G_2 _checkpoint function with aging *in vitro*. For these cells at PDL 70 – 80, the mitotic index in irradiated cells was about half of that seen in sham-treated controls. The telomerase-negative, p53-H179Q line displayed a more modest decrement of G_2 _checkpoint function during aging with at most 17% of cells evading G_2 _delay. This represents the first example in a total of seven independent analyses of p53-defective human fibroblasts [6,25,37] in which G_2 _checkpoint function did not appear to be severely attenuated in cells at crisis. Interestingly the G_2 _checkpoint response of the two spontaneously derived immortal lines (F1-HIT+p53-A143V Immortal and F1-HIT+p53-H179Q Immortal) was very similar to the younger (precrisis) parental lines (Figure 7).

In contrast to the aging-associated attenuation of G_2 _checkpoint function in the telomerase-negative, p53-defective lines, there was no aging-associated attenuation of G_2 _checkpoint function in the telomerase-expressing, p53-defective lines. Cells at >150 PDL displayed a response to IR that was nearly equivalent to that seen at PDL 30 – 40. Thus, the age-related attenuation of G_2 _checkpoint function in p53-defective lines was prevented entirely by expression of telomerase.

### Cytogenetics of immortal lines

Spontaneously immortalized cells emerged from crisis after three months in culture (F1-HIT+p53-A143V Immortal and F1-HIT+p53-H179Q Immortal cell lines). These two cell lines had DNA damage G_1 _and G_2 _checkpoint responses that were similar to those seen in their low-passage parents (Table 2 and Figure 7, respectively). G-band karyotype analysis of these immortal cell lines revealed that both were near diploid (45 – 46 chromosomes, Table 4). The F1-HIT+p53-H179Q immortal line exhibited few abnormalities (12% 18q+, 8% 18q-) with 92% of metaphases being 45 XY or 46 XY. The F1-HIT+p53-A143V immortal line exhibited a somewhat reduced number of diploid metaphases (72%) and an increased number of polyploid metaphases (20%) as well as a small deletion at 4p16 in greater than 50% of metaphases. The lines that were transduced with hTERT when at low PDL were maintained in culture for greater that 175 population doublings. Up to this point, the telomerase positive, p53-defective lines displayed a normal diploid karyotype with no marker chromosomal aberrations (Table 4). However, the F1-hTERT+LXIN control line exhibited a trisomy for chromosome 8 in 92% of metaphases. Interestingly the F1-hTERT+LXIN cell line had a normal karyotype (25 of 25 metaphases) at an intermediate PDL of 96.

**Table 4:** Karyotypic Analysis of Cell Lines

**Cell Line**	**Chromosome Number**^**1**^	**Karyotype**^**2**^
F1-hTERT+LXIN (^3^PDL 173.5)	46, XY (16%)	+8 [72%]
	47, XY (76%)	+8, -Y [8%]
		+8, -16
		+8, -11
		+8, 5q-

F1-hTERT+p53-A143V (PDL 217.6)	46, XY (80%)	t(1;4)
		12q-
		-13
		-X
		-15
		+ marker

F1-hTERT+p53-H179Q (PDL 200.8)	46, XY (84%)	+20
		12p-
		15q-
		19q-
		-7
		+ marker (iso 5p?)
		-21

F1-hTERT+E6 (PDL 160.2)	46, XY (80%)	-Y
		-Y, 11q-
		-6, 12p-
		-15
		+4
		9q chromatid break

F1-HIT+p53-A143V Immortal	45, XY (16%)	4p- [24%]
	46, XY (56%)	4p-, 4q- [16%]
	>90 (20%)	two 4p-, two 4q-, dic 13q;13q, four of every chromosome
		-Y
		6q-
		-13, 4p+
		-15, -18, 3p-, 2 markers
		-15, -18, 3p-
		-19, 4p-
		4p-, dic 2p;2p
		-14, -15, t(5q;14q), t(4p;15q)
		-22, 4p-
		-20, 10p+
		seven 22+

F1-HIT+p53-H179Q Immortal	45, XY (20%)	-4, 18q-
	46, XY (72%)	4p+, -11, -16, 18q-
		18q+ [8%]
		16q-
		-21, -22, 18q+
		-18, 4p+
		-15
		-17
		-18
		4q chromatid break

1. Twenty five metaphases analyzed. Number in parenthesis is percentage of metaphases with given number of chromosomes.

2. Karyotype of samples. Number in brackets is percentage of metaphases examined with given karyotype.

3. PDL, population doubling level. PDL of the spontaneously immortalized lines cannot be determined due to the extended period of time the cells spent in crisis.

## Discussion

Induced expression of telomerase at the time of inactivation or attenuation of p53 function fully blocked the chromosomal destabilization that is commonly associated with defective p53 in human cells. Thus aneuploidization, polyploidization, and formation of chromosomal aberrations including rings, dicentrics, breaks and exchanges, all of which develop in human cells with inherited or transduced mutant p53 alleles [38,39] or after transduction of viral oncoproteins that inactivate p53 [40-42] appear to be secondary consequences of telomere erosion. Telomerase-expressing fibroblasts with little or no p53-dependent G_1 _checkpoint function exhibited no detectable numerical or structural chromosomal alterations after being carried through >170 population doublings. This finding has implications for the mechanisms of genetic instability in cancer and its precursors.

In this study eight isogenic cell lines were derived that differed from each other by one or two alleles. All cell lines expressing dominant-negative forms of p53 or the HPV16E6 oncoprotein to interfere with p53 signaling bypassed the senescence checkpoint. However, expression of the p53-A143V allele caused only a 50 % attenuation of DNA damage G_1 _checkpoint function regardless of telomerase expression. The partial expression of this checkpoint response was associated with partial induction of p21^Waf1 ^protein 6 hr after exposure to 1.5 Gy IR (Figure 3, and Figure 5). This observation is consistent with the report that the V143A allele of p53 binds to and transactivates p53-responsive promoters [43]. When transduced by transfection the A143V allele of p53 has been reported to override the senescence checkpoint and induce severe chromosomal destabilization as seen here [44].

The continued erosion of telomeres in cells bypassing the replicative senescence checkpoint results in a cycle of formation of dicentric chromosomes leading to non-disjunction errors and broken chromosomes that, when repaired, may result in translocations, deletions, or more dicentrics. The telomerase-expressing p53 defective cells used in this study accumulated no structural or numerical abnormalities when grown in culture for greater than 170 population doublings. In contrast to other reports, the telomerase-positive control cell line (F1-hTERT+LXIN) demonstrated no evidence of chromosomal destabilization or morphological changes through approximately 100 PD's [14,45]. However during the next 75 population doublings this cell line did acquire a trisomy for chromosome 8. The trisomy for chromosome 8 seen in the F1-hTERT+LXIN cells at PDL 175 has also been observed in this laboratory in another telomerized human fibroblast line around PDL 200 [46]. Trisomy for chromosome 8 is commonly found in many hyperproliferative disorders and is believed to provide a growth advantage to those cells that acquire it [47-50] Such a growth advantage could have selected for the cell or cells that underwent nondisjunction and acquired the extra chromosome, although population expansion by the F1-hTERT+LXIN line did not increase after PDL 100 (Figure 4B). Recent studies demonstrate that the forced expression of telomerase in skin fibroblasts and other cell types enhances cell proliferation [9]. These effects of telomerase did not appear to alter cellular responses to IR-induced DNA damage as shown here or UV-induced DNA damage as previously reported [51].

Chromosomal destabilization associated with telomere crisis has also been correlated with attenuation and inactivation of DNA damage G_2 _checkpoint function [6,25,29]. Because this checkpoint blocks mitosis by cells with damaged chromatids, it was hypothesized that chromosomally unstable cells with defects in the G_2 _checkpoint would have a growth advantage and accumulate in cultures because of selection. An alternative explanation is the severe instability of chromosomes at crisis impeded progression through mitosis, so that upon irradiation damaged cells complete mitosis at a slower than normal rate. Yang *et al*. described a *rad9*-dependent checkpoint in *S. cerevisiae *that responds to the presence of a dicentric chromosome to delay progression through mitosis [52]. If human fibroblasts with dicentric chromosomes are also delayed in their progression through mitosis, this phenomenon could explain the attenuation of G_2 _checkpoint function seen as p53-defective cells enter crisis. As the efficacy of G_2 _checkpoint function is quantified by the degree of emptying of the mitotic compartment 2 h after irradiation, a reduced rate of progression through mitosis to G_1 _will have the effect of sustaining a higher mitotic index in control and irradiated cells. This explanation would view the attenuation of G_2 _checkpoint function during telomere crisis as another manifestation of chromosomal instability. The correction of G_2 _checkpoint function in the immortal lines that emerged from crisis with stable chromosomes also supports this explanation. There are other ways to attenuate DNA damage G_2 _checkpoint function such as inactivation of ATM [53], BRCA1 and 14-3-3ε [54] or overexpression of cyclin B1/Cdk1 kinase [55]. The common attenuation of DNA damage G_2 _checkpoint function in small cell lung cancer but not squamous cell carcinoma [54] implies that this pathway of DNA damage response protects against carcinogenesis in selected targets.

Human fibroblasts with inherited mutations in p53 or ectopic expression of HPV16E6 spontaneously immortalize at a low frequency [6,38]. During crisis, prior to immortalization, these cell lines exhibit both numerical and structural chromosomal abnormalities. The loss of chromosomes (Table 3) seen in these lines may be due to non-disjunction errors or artifacts associated with the processing of the metaphase preparations. It is not possible to distinguish between these possibilities. However, since this was not observed in the telomerized cell lines or in the non-telomerized cell lines prior to crisis, this phenomenon is likely to be a function of the underlying chromosomal instability. The cell lines, which result from spontaneous immortalization, have usually activated telomerase expression although in some cases telomeres appear to be stabilized by an alternative mechanism (ALT) [56]. These immortal lines are often aneuploid [38]. In this study, the F1-HIT+p53-A143V and F1-HIT+p53-H179Q lines yielded immortal derivatives. These immortal lines were both diploid (45 – 46 chromosomes). The p53-A143V line displayed a marker chromosomal aberration (4p-) in over half of the metaphases. Deletions in this region are associated with Wolf-Hirschhorn syndrome [57]. The p53-H179Q immortal line primarily contained aberrations in chromosome 18 (both losses and gains). Chromosome 18 aberrations have been associated with various malignancies in humans [58,59]. These findings demonstrating only subtle changes in the karyotype following immortalization are consistent with the previous observation that aneuploidy is not an inevitable outcome of *in vitro *immortalization [60]. Taken together, these results may reflect the fact that all that is required for continuous *in vitro *growth of human fibroblasts is hTERT expression. Genetic changes that are not cytogenetically detectable apparently are able to derepress hTERT expression and induce immortality.

## Authors' contributions

DAS constructed cell lines, carried out checkpoint analysis, participated in karyotype studies, study design, and drafted manuscript. EL carried out the G-banding of chromosomes and fine karyotypic analysis. TPH carried out the Western blot analysis of p53 and p21 induction. WKK was instrumental in study design and manuscript preparation.
